# A Robust Adaptive Mesh Generation Algorithm: A Solution for Simulating 2D Crack Growth Problems

**DOI:** 10.3390/ma16196481

**Published:** 2023-09-29

**Authors:** Abdulnaser M. Alshoaibi, Yahya Ali Fageehi

**Affiliations:** Mechanical Engineering Department, College of Engineering, Jazan University, KSA, 114 Almarefah Rd., Jizan 45142, Saudi Arabia

**Keywords:** adaptive mesh generation, crack growth, SIFs, finite element analysis, mesh smoothing and refinement

## Abstract

This paper introduces a robust algorithm that efficiently generates high-quality unstructured triangular meshes to model complex two-dimensional crack growth problems within the framework of linear elastic fracture mechanics (LEFM). The proposed Visual Fortran code aims to address key challenges in mesh generation including geometric complexity, required simulation accuracy, and computational resource constraints. The algorithm incorporates adaptive refinement and updates to the mesh structure near the crack tip, resulting in the formation of rosette elements that provide accurate approximations of stress intensity factors (SIFs). By utilizing the maximum circumferential stress theory, the algorithm predicts the new crack path based on these SIFs. Throughout the simulation of crack propagation, a node splitting approach was employed to represent the progression of the crack, while the crack growth path is determined by successive linear extensions for each crack growth increment. To compute stress intensity factors (SIFs) for each increment of crack extension, a displacement extrapolation method was used. The experimental and numerical results demonstrated the algorithm’s effectiveness in accurately predicting crack growth and facilitating reliable stress analysis for complex crack growth problems in two dimensions. The obtained results for the SIF were found to be consistent with other analytical solutions for standard geometries.

## 1. Introduction

The computational design of structural materials with existing cracks necessitates a crucial step: reliably assessing their remaining service life. In order to predict phenomena such as fatigue life, simulating crack growth becomes essential, typically involving a LEFM problem at each load step. The study of fracture behavior involves a combination of laboratory experiments, analytical investigations, and numerical simulations. While experiments are necessary to some degree, they tend to be expensive and time consuming [1]. Analytical methods are convenient but have limitations in handling complex configurations and loads due to the challenges of mathematical derivation [2,3]. In situations where engineering structural parts exhibit intricate geometries, material properties, and loading conditions, numerical simulations are commonly employed to analyze the propagation of static and fatigue cracks. Various numerical methods have been proposed by scholars to address the issue of crack propagation. These methods include the finite element method (FEM), extended finite element method (XFEM), meshless method, meshfree method, boundary element method, and phase field model. The FEM is a highly versatile numerical method widely used for simulating scenarios with discontinuities. Nevertheless, when it comes to simulating crack growth, a notable challenge arises in the spatial discretization of the domain, commonly referred to as the mesh generation. Continual adaptation and evolution of the mesh generation process at each load step are essential to accurately capture the evolving topology of cracks [4,5,6]. This dynamic approach ensures that the mesh effectively represents the changing geometry of the cracks as they propagate and grow during the simulation [7]. The field of fracture mechanics in computational studies encounters major challenges associated with the bottleneck issues of automatic mesh generation and adaptation. Mesh generation is a crucial element encompassing multiple important considerations such as grid quality, efficiency, flexibility, level of automation, and robustness. These factors play a crucial role in addressing the constraints within this field. Simulating crack propagation using the FEM involves various distinct approaches that offer different advantages and capabilities. These methods include the utilization of partition of unity method [8,9], nodal release at the crack tip [10,11], remeshing at a global or local level after each crack growth step [12,13], and the implementation of strain and/or displacement enrichment [14]. These techniques serve as the basis for advanced methods such as the XFEM [15,16] or phase-field crack modeling [17,18]. Mesh generation is a fundamental step in numerical simulations, essential for accurately representing the geometry and topology of computational domains. Various methods have been developed to generate meshes for different types of applications, ranging from simple geometries to complex structures. Two well-known mesh generation techniques are the advancing front method [19,20,21] and the Delaunay method [22]. These methods have been widely employed in diverse fields, including computational fluid dynamics, structural analysis, and finite element simulations. The advancing front method is a popular technique used to generate unstructured meshes. It involves dividing the domain into smaller regions using an advancing front that progressively moves throughout the boundary. The method employs a front of triangular or quadrilateral elements that expands and grows as new nodes are added. This method provides good control over mesh quality and is particularly suited for domains with complex geometries and irregular boundaries. The Delaunay method is a widely used algorithm for generating triangular meshes. It is based on the Delaunay triangulation concept, ensuring that no point lies within the circumcircle of any triangle in the mesh. The Delaunay method produces high-quality meshes with well-shaped elements that satisfy certain optimality criteria. It is particularly effective for two-dimensional domains and finds applications in computational geometry, finite element analysis, and computer graphics. The accuracy of a finite element analysis model is directly influenced by the refinement of the finite element mesh. One simple method for refining a mesh is by uniformly reducing the element size across the entire modeling domain. This approach is straightforward but lacks the ability to selectively refine specific regions that require a finer mesh. One of the primary objectives of mesh refinement is to strike a balance between computational efficiency and solution accuracy. While a highly refined mesh provides more accurate results, it also increases the computational cost and time required to perform simulations. Therefore, achieving an optimal level of mesh refinement is essential to achieve accurate results within practical limitations. Researchers have been continuously seeking new and improved crack growth algorithms based on the FEM for decades. Advances in computational power and modeling techniques have allowed them to develop ever more sophisticated simulations of crack propagation. Wang et al. [23] introduced a multifunctional super singular element-based adaptive FEM for crack growth. The crack propagation algorithm employed in their study achieved automatic parametric modeling by utilizing Python programming language for secondary development of the pre-processing module in ABAQUS. Areias et al. [24] introduced a crack growth algorithm that is not reliant on specific constitutive laws or element technology. This algorithm enables the accurate capture of strain localization, even in cases where the initial mesh is not fine enough. Another algorithm for minimizing the area/volume of damaged elements to improve the representation of crack paths was proposed by Areias et al. [25]. Their approach involves a combination of a staggered algorithm, a modified screened Poisson equation, and adaptive mesh refinement with mesh contraction capabilities. The crack propagation algorithms mentioned earlier [24,25] were observed to lack the incorporation of fracture mechanics analysis at the crack tip or specific criteria for crack propagation. Accurate prediction of the stress intensity factor (SIF) requires the inclusion of mesh refinement in the vicinity of the crack tip to accommodate crack growth. It is necessary to continuously modify the finite element meshes to ensure that the refined meshes are consistently positioned at the crack tip [26,27]. Consequently, during crack propagation, partial or global remeshing becomes necessary. To strike a balance between computational accuracy and efficiency, researchers employ diverse procedures. These approaches encompass algorithm selection, mesh refinement, adaptive methods, and optimization of numerical parameters. By carefully implementing these techniques, researchers can achieve the desired level of accuracy in simulations while optimizing computational resources and reducing computational costs. One commonly used technique for modeling crack propagation is the “debonding technique” [28], where crack growth is simulated by disconnecting the shared node between two elements, effectively representing the separation and propagation of the crack. This restricts the crack to propagate only along element edges in the mesh. As a result, dense meshes are required to accurately capture the crack path when using this technique. In the context of simulating crack growth, Park et al. [29] utilized partial mesh reconstruction techniques for adaptive mesh refinement (or coarsening). Additionally, Khoei et al. [30] introduced a global adaptive remeshing procedure for simulating crack growth in two-dimensional elastic materials. Implementing these advanced techniques for crack growth simulations, which yield more accurate results, requires proficient programming skills to develop functional codes. The utilization of these techniques necessitates expertise in programming and a deep understanding of the underlying algorithms to effectively incorporate them into the simulation framework [31]. To ensure accurate and reliable results, it is crucial to maintain well-structured meshes in the crack tip regions when employing adaptive meshing approaches. Poorly shaped elements with unfavorable aspect ratios can have a negative impact on the quality of the solution [32]. Therefore, it remains crucial to develop a model that ensures accurate solutions for the stress field at the crack tip and provide an efficient strategy for adaptive mesh refinement during crack propagation. However, accomplishing this task is currently challenging and can be addressed by only a limited number of finite element codes. The development of software capable of automating the crack growth process would be highly desirable. Such software would streamline and simplify the simulation of crack propagation, enabling efficient and reliable analysis without requiring extensive manual intervention. This study focuses on the development of source code using the Visual Fortran programming language for mesh generation in the context of crack growth simulation, with the objectives of creating a flexible and automated mesh generation algorithm capable of simulating crack growth and providing accurate results for subsequent analysis. Through the utilization of Visual Fortran’s computational capabilities, efficient and reliable mesh generation can be achieved, facilitating an accurate crack growth simulation and supporting the assessment of structural components and materials. The main purpose of this paper is to highlight the theoretical background that underlies the development of source code used for generating two-dimensional meshes, which are essential for simulating the process of crack growth. By focusing on the theoretical aspects, the study aims to offer a comprehensive comprehension of the fundamental principles and concepts essential for creating specialized source code to generate meshes specifically tailored for simulating fatigue crack growth. The present study provides insights into various aspects that were not explicitly mentioned in the previous work of the author in reference [33]. These include the generation front, which involves the generation of the initial crack front geometry, as well as mesh smoothing techniques to improve the quality of the finite element mesh. Additionally, the manuscript discusses the smoothing stresses method, which enhances the accuracy of stress calculations and delves into the representation of shape functions. Furthermore, the effect of mesh refinement on the results of stress intensity factors is also explored in this review article. This analysis sheds light on the impact of mesh density on the accuracy and reliability of stress intensity factor calculations, providing valuable insights for researchers and practitioners in the field. Additionally, the primary focus of the article is to evaluate the fidelity of the crack growth simulations performed using the developed mesh generator, analyzing the accuracy of crack growth predictions, the ability to capture crack behavior under different loading conditions, and the reliability of the simulation results compared to experimental or analytical data.

## 2. Developed Program Procedure

### 2.1. Analytical Solution of the Plane Crack Problem

**Figure 1:**
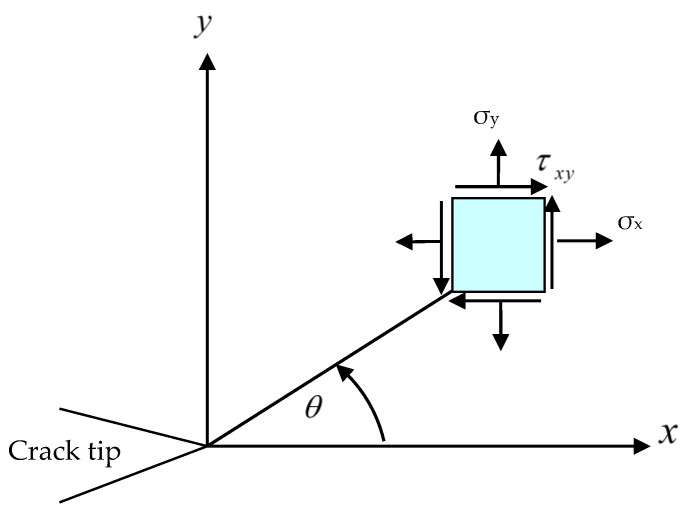
The stress element in polar coordinate system.

With reference to Figure 1, the equilibrium equations for plane elasticity in Cartesian coordinates can be analyzed as

(1) $$\begin{array}{c} \frac{\partial \sigma_{x}}{\partial x}+\frac{\partial \tau_{xy}}{\partial y}=0\\ \frac{\partial \sigma_{y}}{\partial y}+\frac{\partial \tau_{xy}}{\partial x}=0 \end{array}$$

The Airy stress function approach is a powerful mathematical tool used in solid mechanics to obtain analytical solutions for stress components $\sigma_{x},\sigma_{y}$ and $\tau_{xy}$. The methodology relies on employing a scalar function called the Airy stress function $\psi \left(x,y\right)$, which meets the requirements of the biharmonic operator $\nabla^{4}\psi =0$.

Using the Airy stress function, the stress components can be expressed as

(2) $$\begin{array}{c} \sigma_{x}=\frac{\partial^{2}\psi }{\partial y^{2}}\\ \sigma_{y}=\frac{\partial^{2}\psi }{\partial x^{2}}\\ \tau_{xy}=-\frac{\partial^{2}\psi }{\partial x\partial y} \end{array}$$

In solid mechanics, the compatibility equation is a crucial requirement that must be met by the strain field in a solid to ensure that it represents a physically realistic deformation. This equation is particularly important in stress formulations, where it relates the normal and shear strains in a solid to each other and to the deformation of the solid as a whole. In the context of stress formulations in solid mechanics, it is necessary to ensure that the strain field in a solid satisfies a compatibility equation as

(3) $$\nabla^{2}\left(\sigma_{x}+\sigma_{y\;}\right)=0$$

in which $\nabla^{2}$ is the usual Laplace or harmonic operator. When Equation (2) is substituted into Equation (3), it leads to the following biharmonic equation:

(4) $$\nabla^{4}\phi \;=\;\nabla^{2}(\nabla^{2}\phi )\;=\;0$$

Typically, a linear elasticity problem involving plane extension can be solved by identifying a stress function that meets the requirements of Equation (4). Additionally, it is necessary to verify that the boundary conditions of the problem are satisfied by the stresses calculated using Equation (2).

The primary objective of LEFM simulations for crack propagation is typically to investigate the combined influence of mode I and mode II. Therefore, researchers are interested in studying how cracks behave under different combinations of tensile and shear stress. There are multiple intricate versions of the Airy stress function that can be employed to address crack problems. In the context of in-plane cracks, Irwin [34] extensively analyzed this mixed-mode problem and employed Westergaard [35] stress functions to derive an analytical solution for the stress distribution near the crack tip as follows:

(5) $$\begin{array}{l} \sigma_{x}\;=\;\frac{K_{I}}{\sqrt{2\pi r}}\cos\frac{\theta }{2}\;\left(1-\sin\frac{\theta }{2}\;\sin\frac{3\theta }{2}\right)\;-\;\frac{K_{II}}{\sqrt{2\pi r}}\;\sin\frac{\theta }{2}\;\left(2\;+\;\cos\frac{\theta }{2}\;\cos\frac{3\theta }{2}\right)\\ \sigma_{y}\;=\;\frac{K_{I}}{\sqrt{2\pi r}}\cos\frac{\theta }{2}\;\left(1+\sin\frac{\theta }{2}\;\sin\frac{3\theta }{2}\right)\;\;+\;\frac{K_{II}}{\sqrt{2\pi r}}\;\sin\frac{\theta }{2}\;\cos\frac{\theta }{2}\;\cos\frac{3\theta }{2}\\ \tau_{xy}\;=\;\frac{K_{I}}{\sqrt{2\pi r}}\cos\frac{\theta }{2}\;\sin\frac{\theta }{2}\;\cos\frac{3\theta }{2}\;\;+\;\;\frac{K_{II}}{\sqrt{2\pi r}}\;\cos\frac{\theta }{2}\left(1-\sin\frac{\theta }{2}\;\sin\frac{3\theta }{2}\right) \end{array}$$

where *r* represents the radial distance from the crack tip, $\sigma_{x},\sigma_{y}$ denotes the stress components in the *X* and *Y* directions, $\tau_{xy}$ represents the shear stress component in the *XY* direction, θ is the crack growth angle, and *K_I_* and *K_II_* correspond to the modes of SIF. 

Subsequently, this solution has gained significant recognition as a fundamental tool in fracture mechanics research. It has empowered scientists to gain valuable insights into the behavior of cracks in diverse materials and structures. The stress distribution is depicted in Figure 1 using the polar coordinate system. Perez [36] (2004) showed that it is possible to relate the displacements to the SIFs, as well as the stresses, using the following expression:

(6) $$\begin{array}{l} u\;\;=\;\;\frac{1}{4\mu }\;\sqrt{\frac{r}{2\pi }}\;\left[K_{I}\;\left((2\kappa -1)\cos\frac{\theta }{2}-\cos\frac{3\theta }{2}\right)\;\;-\;\;K_{II}\;\left((2\kappa +3)\sin\frac{\theta }{2}+\sin\frac{3\theta }{2}\right)\right]\\ v\;\;=\;\;\frac{1}{4\mu }\;\sqrt{\frac{r}{2\pi }}\;\left[K_{I}\;\left((2\kappa +1)\sin\frac{\theta }{2}-\sin\frac{3\theta }{2}\right)\;\;-\;\;K_{II}\;\left((2\kappa -3)\cos\frac{\theta }{2}+\cos\frac{3\theta }{2}\right)\right] \end{array}$$

where μ is the shear modulus of the material; *ν* denotes the Poisson’s ratio; *K_I_* and *K_II_* are the first and second mode of stress intensity factor, respectively; *θ* denotes the crack growth angle; and κ is given by

(7) $$\kappa =\left\{\begin{array}{c} 3-4\nu \;\;\;\left(for\;plane\;strain\;condition\right)\\ \frac{3-\nu }{1+\nu }\;\;(for\;plane\;stress\;condition) \end{array}\right.$$

### 2.2. Isoparametric Formulation of Quadratic Shape Functions

**Figure 2:**
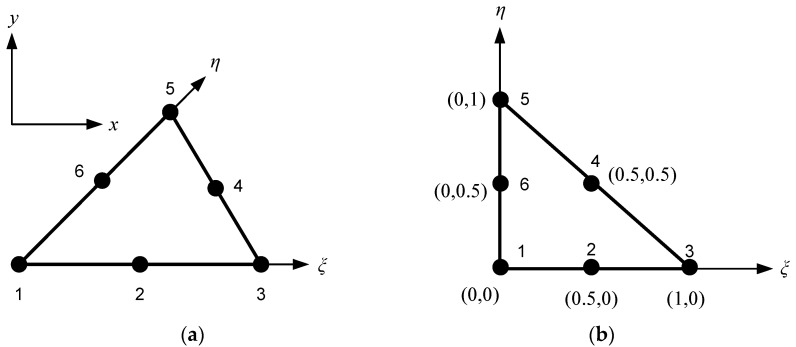
A quadratic triangle element: (**a**) global coordinates; (**b**) natural coordinates.

Isoparametric quadratic triangular shape functions find common usage in the analysis of structures and other physical systems that demand accurate modelling. This technique involves using the same set of coordinates to describe both the geometry of the element and the variation of the field variable within the element. The accuracy and efficiency of a given element type in finite element analysis depends on the capability of its shape functions to accurately represent the true displacement field. The isoparametric family of elements is a group of elements that employ shape functions for both defining the geometry and the displacement field. In the current study, a quadratic triangle element with quadratic shape functions was utilized as a higher-order element to achieve a more accurate representation of the displacement field within the element. This element type requires six nodes, with three nodes located at the corners of the triangle and three additional nodes located at the midpoints of each side. The additional nodes enable the element to accurately capture the curvature of the displacement field within the element. Figure 2 illustrates the representation of this element in both the global coordinate system $\left(x,y\right)$ and the natural coordinate system $\left(\xi ,\eta \right)$.

The shape functions for each node are given by the following equation:

(8) $$\begin{array}{l} N_{1}=\left(1-\xi -\eta \right)\left(1-2\xi -2\eta \right)\\ N_{2}=\xi \left(2\xi -1\right)\\ N_{3}=\eta \left(2\eta -1\right)\\ N_{4}=4\xi \left(1-\xi -\eta \right)\\ N_{5}=4\xi \eta \\ N_{6}=4\eta \left(1-\xi -\eta \right) \end{array}$$

The coordinate values $x\left(\xi ,\eta \right)$ and $y\left(\xi ,\eta \right)$ at any point $\left(\xi ,\eta \right)$ within the element can be defined as

(9) $$\begin{array}{l} x\left(\xi ,\eta \right)=\sum_{i=1}^{6}N_{i}\left(\xi ,\eta \right)x_{i}\\ y\left(\xi ,\eta \right)=\sum_{i=1}^{6}N_{i}\left(\xi ,\eta \right)y_{i} \end{array}$$

where $\left(x_{i},y_{i}\right)$ denotes the coordinates of node *i*. Likewise, the calculation of displacements can be performed using relevant equations such as

(10) $$\begin{array}{l} u\left(\xi ,\eta \right)=\sum_{i=1}^{6}N_{i}\left(\xi ,\eta \right)u_{i}\\ v\left(\xi ,\eta \right)=\sum_{i=1}^{6}N_{i}\left(\xi ,\eta \right)v_{i}. \end{array}$$

The evaluation of the Jacobian matrix, which will be needed later, is performed as follows:

(11) $$J^{e}\;=\;\left[\begin{array}{cc} \frac{\partial x}{\partial \xi } & \frac{\partial y}{\partial \xi }\\ \frac{\partial x}{\partial \eta } & \frac{\partial y}{\partial \eta } \end{array}\right]\;=\sum_{i=1}^{6}\left[\begin{array}{cc} \frac{\partial N_{i}}{\partial \xi }\;\cdot x_{i} & \frac{\partial N_{i}}{\partial \xi }\;\cdot y_{i}\\ \frac{\partial N_{i}}{\partial \eta }\;\cdot x_{i} & \frac{\partial N_{i}}{\partial \eta }\;\cdot y_{i} \end{array}\right]\;$$

The expression for the inverse of the Jacobian matrix is as follows:

(12) $${\left[J^{e}\right]}^{-1}\;=\;\left[\begin{array}{cc} \frac{\partial \xi }{\partial x} & \frac{\partial \eta }{\partial x}\\ \frac{\partial \xi }{\partial y} & \frac{\partial \eta }{\partial y} \end{array}\right]\;=\;\frac{1}{\det\;J^{e}}\;\;\left[\begin{array}{cc} \frac{\partial y}{\partial \eta } & -\frac{\partial y}{\partial \xi }\\ -\frac{\partial x}{\partial \eta } & \frac{\partial x}{\partial \xi } \end{array}\right].$$

The expression for the discretized elemental volume is provided as follows:

(13) $$dV\;=\;t^{e}\;\det\;J^{e}\;d\xi \;d\eta$$

The value of $t^{e}$ represents the element thickness in the context of plane stress conditions, and its value varies depending on the specific element being considered. On the other hand, for plane strain conditions, its value is fixed at one. The discretized elemental volume is an important factor in FEM, as it is used to calculate various properties of the element, such as mass and stiffness.

The relationships between strain and displacement are mathematically expressed as

(14) $$\varepsilon^{e}\;=\;\sum_{i=1}^{r}B_{i}^{e}\;d_{i}^{e}$$

where $B_{i}^{e}$ denotes the strain matrix and $d_{i}^{e}$ represents the nodal displacement. Both of these quantities are defined in Table 1 for the plane stress and plane strain condition.

The strain displacement matrix provided in Table 1 requires the Cartesian derivatives of the shape functions. These derivatives can be calculated using the chain rule of differentiation and are given by the following expression:

(15) $$\frac{\partial N_{i}}{\partial x}\;=\;\frac{\partial N_{i}}{\partial \xi }\frac{\partial \xi }{\partial x}+\frac{\partial N_{i}}{\partial \eta }\frac{\partial \eta }{\partial x}$$

and

(16) $$\frac{\partial N_{i}}{\partial y}\;=\;\frac{\partial N_{i}}{\partial \xi }\frac{\partial \xi }{\partial y}+\frac{\partial N_{i}}{\partial \eta }\frac{\partial \eta }{\partial y}$$

The values of $\frac{\partial \xi }{\partial x},\;\frac{\partial \eta }{\partial x},\;\frac{\partial \xi }{\partial y}$, and $\frac{\partial \eta }{\partial y}$ can be determined from the inverse of the Jacobian matrix expressed in Equation (12).

### 2.3. Normal and Tangential Load Representation

**Figure 3:**
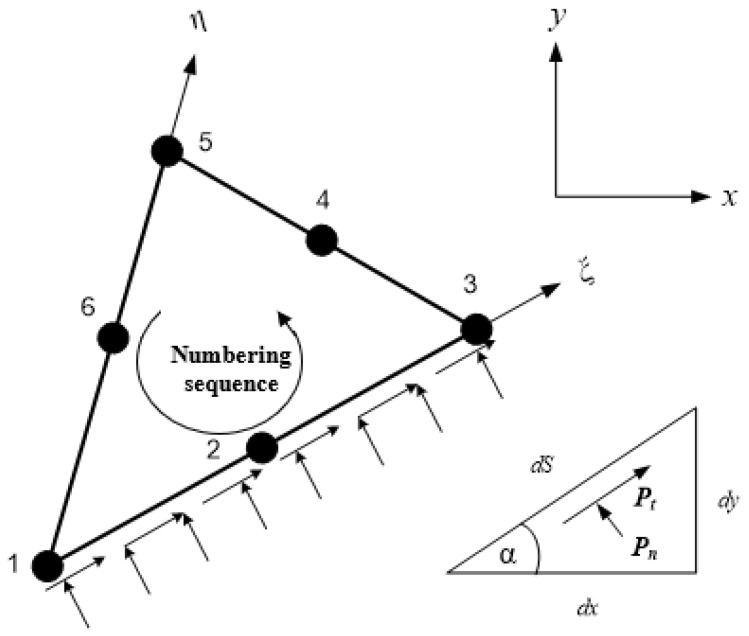
Normal and tangential loads per unit length on quadratic isoparametric triangle elements.

Figure 3 displays a depiction of an element edge that can experience a distributed loading per unit length in both the normal and tangential directions. The type of loading distribution utilized is dependent on the degree of the isoparametric polynomial employed. In this study, quadratic isoparametric elements were utilized, and as such, a quadratic loading distribution was considered the most appropriate. The established convention in this analysis assumed that a load applied normal to a face is considered positive when it acts in the direction into the element. Similarly, a tangential load is regarded as positive when it acts in an anticlockwise direction relative to the loaded element. These assumptions are essential in the analysis of the loaded element and must be considered during the calculation of forces and moments.

The intensity of the distributed loading at any point along the loaded edge is expressed as

(17) $$\left[\begin{array}{c} p_{n}\\ p_{t} \end{array}\right]=\sum_{i=1}^{3}N_{i}\left[\begin{array}{c} {\left(p_{n}\right)}_{i}\\ {\left(p_{t}\right)}_{i} \end{array}\right]$$

where ${\left(p_{n}\right)}_{i}$ and ${\left(p_{t}\right)}_{i}$ represent the nodal values of normal and tangential distributed loads, respectively, with *i* ranging from 1 to 3. On the other hand, $N_{i},\;\;i=1..3$ denotes the three shape functions that define a parabolic variation along the element edge with the following formulae:

(18) $$\begin{array}{l} N_{1}=-\frac{1}{2}\xi \left(1-\xi \right)\\ N_{2}=\left(1-\xi \right)\left(1+\xi \right)\\ N_{3}=\frac{1}{2}\xi \left(1+\xi \right) \end{array}$$

The calculations for the *x* and *y* components of the force acting on an incremental length of the loaded edge are as follows:

(19) $$\begin{array}{l} dP_{x}=\left(p_{t}\;dS\;\cos\;\alpha \;-\;p_{n}\;dS\;\sin\;\alpha \right)=\left(p_{t}\;dx-p_{n}\;dy\right)\\ dP_{y}=\left(p_{n}\;dS\;\cos\;\alpha \;+\;p_{t}\;dS\;\sin\;\alpha \right)=\left(p_{n}\;dx+p_{t}\;dy\right) \end{array}$$

To perform integration along the element edge using the curvilinear variable ξ, the equations provided earlier can be expressed in the following form:

(20) $$\begin{array}{l} dP_{x}=\left(p_{t}\frac{\partial x}{\partial \xi }-p_{n}\frac{\partial y}{\partial \xi }\right)d\xi \\ dP_{y}=\left(p_{n}\frac{\partial x}{\partial \xi }+p_{t}\frac{\partial y}{\partial \xi }\right)d\xi \end{array}$$

Solving these equations provides the expressions for the equivalent nodal forces as

(21) $$\begin{array}{l} P_{xi}=\int_{S_{e}}N_{i}^{e}\left(p_{t}\frac{\partial x}{\partial \xi }-p_{n}\frac{\partial y}{\partial \xi }\right)d\xi \\ P_{yi}=\int_{S_{e}}N_{i}^{e}\left(p_{n}\frac{\partial x}{\partial \xi }+p_{t}\frac{\partial y}{\partial \xi }\right)d\xi \end{array}$$

### 2.4. Automatic Mesh Generation

The process of mesh generation is intricate and prone to errors, particularly when dealing with practical scientific and engineering computations that involve complex geometries of varying complexity. Zienkiewicz and Phillips [38] initiated the development of fully automatic mesh generators that require minimal input to generate valid finite element meshes over arbitrary domains. Since then, many methodologies and algorithms have been proposed to improve the efficiency and accuracy of automatic mesh generation. The construction of the background mesh in the crack propagation program utilizes the dichotomy approach. The proposed approach entails constructing the mesh in a triangular form, incorporating all the initial exterior boundary nodes of the figure. This method represents the computational space as a polygon, progressively splitting the computational domain into two subsets until complete polygon subsets are formed. Further details regarding this approach can be found in [33]. The advancing front method is a popular technique for generating high-quality meshes in two or three dimensions. This method involves dividing the domain into subdomains using a front that advances from the boundary towards the interior of the domain. The front is composed of edges that connect vertices, and the vertices are added to the front as it advances. The advancing front method works by first dividing the boundary into a set of edges, which are connected to form a front. The front is then advanced by adding vertices to the front and connecting them to the existing edges. As new vertices are added, the front is split into smaller subfronts, and each subfront is processed independently. To ensure that the resulting mesh is of high quality, the advancing front method uses several techniques, including edge-flipping and local optimization. Edge-flipping involves swapping the endpoints of an edge to improve the quality of the mesh elements. Local optimization involves adjusting the position of the vertices to improve the quality of the mesh elements in the immediate vicinity. One of the advantages of the advancing front method is that it can handle complex geometries with concave boundaries, which can be challenging for other mesh generation techniques. 

Furthermore, the method can be employed to generate both structured and unstructured meshes, depending on the simulation requirements. The mesh generation procedure typically encompasses three primary steps, as outlined by Zienkiewicz and Taylor [20]:

- Node generation: In the first step, nodes are generated along the boundary edges to create a discretized boundary for the domain.
- Element and node generation: Once the boundary is discretized, the next step is to generate elements and nodes within the boundary.
- Element shape enhancement: In the final step, the quality of the mesh is improved by enhancing the shape of the elements. Element shape refinement techniques involve adjusting the position of the nodes to improve the shape and quality of the elements, such as reducing element distortion or improving element aspect ratios.

### 2.5. Generation Front

**Figure 4:**
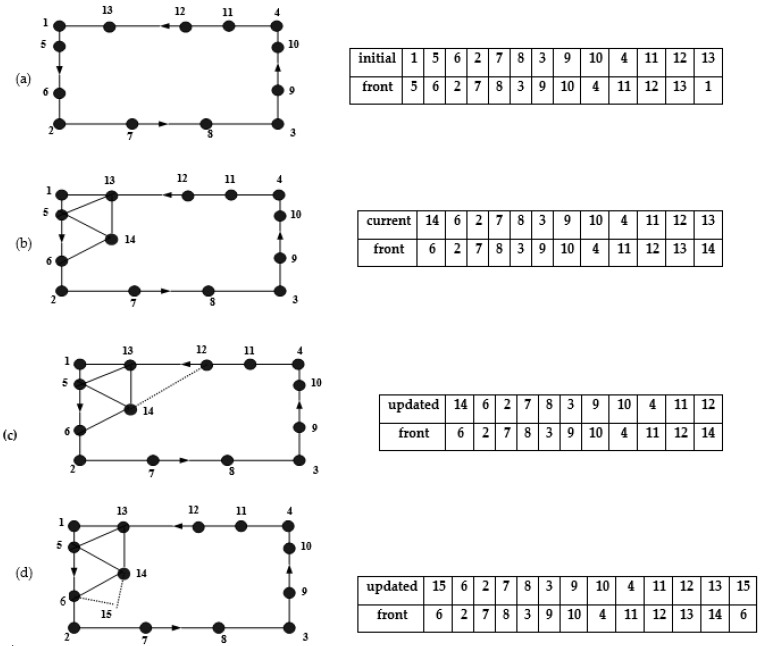
Evolution of the generation front: (**a**) the initial generation front; (**b**) the generation front at a specific stage; (**c**,**d**) updated generation front after creating a new element.

To prepare for the generation of triangular elements, a generation front is created using the discretized boundary edges of the domain. This front is composed of closed loops of boundary sides, and if the domain contains multiple connected regions with different material properties, separate initial fronts are formed for each region. The generation front consists of sides defined by their two endpoints and is arranged in a sequence that ensures the regions to be meshed are always situated to the left of the front. In Figure 4a, the initial generation front of a basic rectangular domain is illustrated. Throughout the element generation process, the generation front consistently outlines the boundary of the region to be meshed, as demonstrated in Figure 4b. During the element generation phase, a side from the generation front is chosen to create a new element with either a newly generated or existing node. Following the formation of a new element, the generation front is updated by removing the utilized side and adding the newly created side, as depicted in Figure 4c,d.

As the front advances, new triangles are generated by connecting adjacent nodes in the front. Each triangle is formed by connecting three nodes in a counterclockwise order. The connectivity of the triangles is based on the specific method used for advancing the front. In their work, Zienkiewicz and Taylor [20] provided a detailed explanation of the process involved in generating elements in a mesh. They outlined the steps involved in generating elements, including domain decomposition, node placement, element connectivity, element refinement, and mesh optimization.

### 2.6. Mesh Smoothing

Mesh smoothing is a post-processing technique employed to enhance the quality of a generated mesh. It involves modifying the positions of nodes and elements to reduce distortions and irregularities present in the mesh. The objective of mesh smoothing is to enhance the accuracy and efficiency of numerical simulations that utilize the mesh. Typically, mesh smoothing entails adjusting the positions of interior nodes while preserving the topological structure of the mesh. This means that the nodal connections of elements remain unaltered, but the interior nodes are repositioned to generate more regular and well-shaped elements. Laplacian smoothing is a well-established and computationally efficient mesh smoothing algorithm, initially introduced by Cavendish [39]. This method entails relocating each node to the average position of its neighboring nodes. The degree of smoothing can be controlled by adjusting the number of iterations and the weighting applied to the node displacements.

The equation utilized to determine the updated position of an internal node *i* through Laplacian smoothing is given by

(22) $$\left(x_{i},y_{i}\right)\;=\;\frac{1}{N}\;\sum_{j=1}^{N}\left(x_{j},y_{j}\right)\;$$

where *N* denotes the number of nodes associated to node *i.*

### 2.7. Smoothing Stresses

In finite element analysis, stresses are typically evaluated at discrete sampling points within the computational domain. To produce a more visually appealing and physically meaningful representation of the stress field, these discrete stresses must be “smoothed” to create a continuous distribution of stresses that can be plotted using contour plots or other visualization techniques. The resulting smoothed stresses are important in the error estimation process, particularly in *h*-type adaptive mesh refinement, where the mesh is refined based on the distribution of the stresses. The choice of smoothing technique and its impact on the accuracy and efficiency of the numerical simulations must be carefully considered. If the sampling points overlap with the mid-side points of isoparametric six-noded elements in finite element analysis, only the stresses at the other three nodes of the element need to be smoothed. To obtain smoothed stresses, the mid-side point stresses can be extrapolated to the other nodes using techniques such as polynomial fitting or averaging. However, it is important to note that the accuracy of the smoothed stresses is contingent upon the quality of the mid-side point stresses.

### 2.8. Adaptive Remeshing

In *h*-type adaptive mesh refinement, the objective is to determine the relative stress norm error, which represents the ratio of the element norm stress error to the average norm stress error across the computational domain. This ratio serves as a guide to predict the necessary mesh refinement size for the subsequent iteration. The element norm stress error quantifies the discrepancy between the computed stress value and the true stress value within a specific element, while the average norm stress error is the mean of the element norm stress errors across the entire domain. The refinement process entails increasing the mesh density in regions where the element norm stress error is relatively high compared to the average norm stress error. This approach can yield significant computational savings by reducing the number of elements required to achieve a desired level of accuracy. The adaptive remeshing method employed in this study is extensively explained in [40,41,42,43,44]. The *h*-type adaptive mesh procedure allows for the definition of the mesh size for each element based on the required level of refinement in a particular region of the problem domain. The following expression is utilized to determine the mesh size of each element:

(23) $$h_{e}=\sqrt{2A_{e}}$$

where *A_e_* represents the area of the triangular element.

### 2.9. Quarter-Point Singular Element

**Figure 5:**
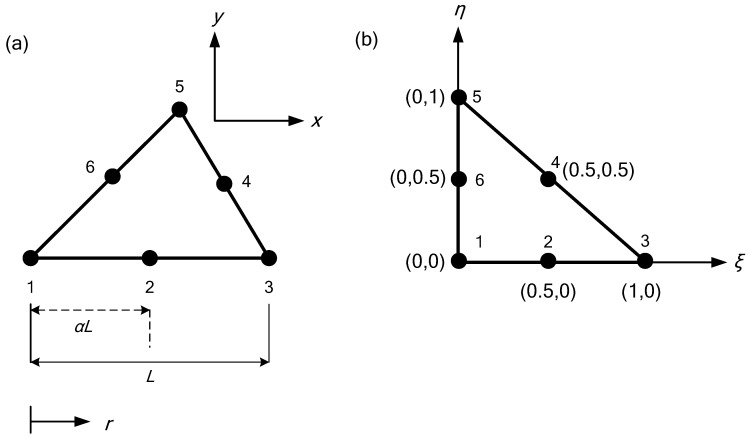
(**a**) Shifting of a mid-side node of triangle by factor of α, and (**b**) isoparametric representation.

In finite element analysis, a singular element is an element that has a zero or near-zero area or volume, resulting in singularities that can cause numerical instabilities or inaccuracies in the solution. The triangle singular element is a type of triangular element that is designed to handle singularities in two-dimensional problems. Consider a six-noded quadratic triangle element (T6) in Cartesian space, as depicted in Figure 5a. The element has a length *L* along the *r* direction, and the crack tip is positioned at *r* = 0.0 (or node 1). The distance of node 2 from the crack tip is adjusted by a factor of α. Figure 5b displays the element representation in parametric space, where the element shape functions are defined.

To determine the displacement *u* at any point within the T6 element, the nodal displacements $u_{i}$ are interpolated using the quadratic shape functions *N* as follows:

(24) $$u=\sum_{i=1}^{6}N_{i}u_{i}$$

**Figure 6:**
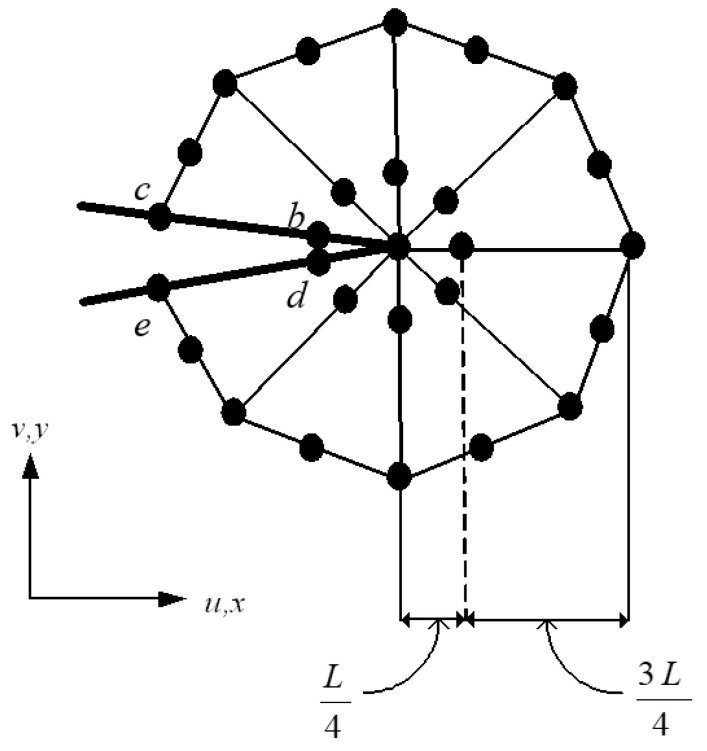
Rosette construction of natural triangle quarter-point elements around a crack tip.

**Figure 7:**
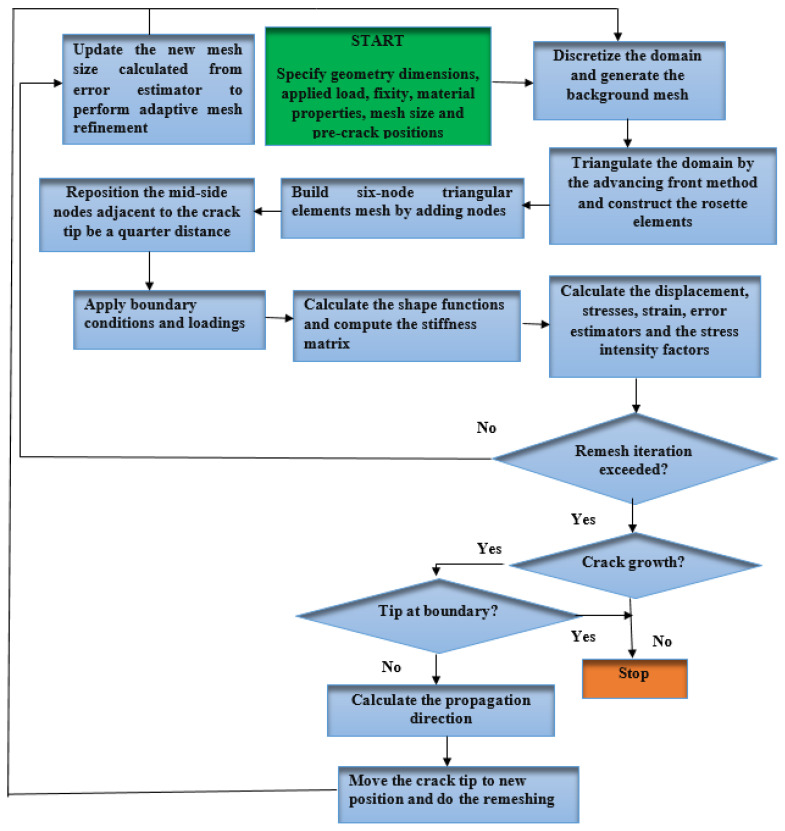
Comprehensive procedure for the crack growth program.

Here, $N_{i}$ refers to the isoparametric triangular element shape functions, which are represented by Equation (8). These functions are used to interpolate the nodal values of the displacement field within the T6 element. To model the linear elastic singularity in the stress and strain fields near the crack tip, natural triangle quarter-point elements, as introduced by Freese and Tracey [45], are used. The number of quarter-point elements plays a crucial role in accurately modeling stress distribution at crack-tips. Utilizing T6 elements and increasing element count enhances the fidelity of stress representation, ensuring a more precise depiction of the stress distribution. In the developed program, the choice of rosette elements is crucial for accurately modeling stress distribution around crack-tips. These rosette elements around the crack tip are available in set configurations, including 6, 8, 12, 14, 20, and 24. Figure 6 depicts a schematic illustration of the formation of rosette patterns using quarter-point elements around a crack tip, where *b,c,d* and *e* are the nodes around the crack tip. The diagram presented in Figure 7 illustrates the computational approach used in the crack propagation program. The crack growth flowchart begins with specifying the geometry dimensions, applied load, fixity, material properties, mesh size, and pre-crack positions. Once these parameters are defined, the domain is discretized, and a background mesh is generated. The next step involves triangulating the domain using the advancing front method and constructing the rosette elements.

After the mesh is prepared, boundary conditions and loadings are applied to simulate the real-world scenario. Shape functions are then calculated, and the stiffness matrix is computed to determine the mechanical response of the structure. Using this information, the displacement, stresses, strains, error estimators, and stress intensity factors are calculated. At this stage, a remesh iteration may be performed to refine the mesh and improve accuracy. This iteration helps to capture the evolving crack geometry more effectively. Finally, based on the calculated results and considering factors such as stress intensity factors, crack growth direction is determined.

The process of releasing nodes based on their mechanical properties, commonly referred to as the “relaxation of split nodes”, is employed to simulate crack growth. In order to accurately represent crack openings when the conditions for crack propagation are met, it is crucial to have two distinct nodes at the crack tip. To visualize the deformation, the displacements need to be appropriately adjusted using the coordinates of the boundary nodes. A comprehensive description of the node splitting and relaxation procedure can be found in a previous study [41]. The stress intensity factors, which are utilized to determine the crack growth direction based on the maximum circumferential stress theory, can be calculated using two different methods. Based on the maximum circumferential stress theory, the crack growth direction is then predicted to be perpendicular to the direction of maximum tensile stress. The first method is the displacement extrapolation method, which is described in detail in [33,46]. The second method is the J-integral method, which is explained comprehensively in [47].

## 3. Numerical Results and Discussions

In this section, a variety of meshes produced by the developed mesh generator are presented as examples. These meshes are intended to showcase the versatility and effectiveness of the mesh generator in handling diverse geometries and boundary conditions. Through these examples, readers can gain a deeper understanding of the capabilities of the mesh generator and its potential applications in various fields, including structural mechanics.

### 3.1. Compact Tension Specimen

**Figure 8:**
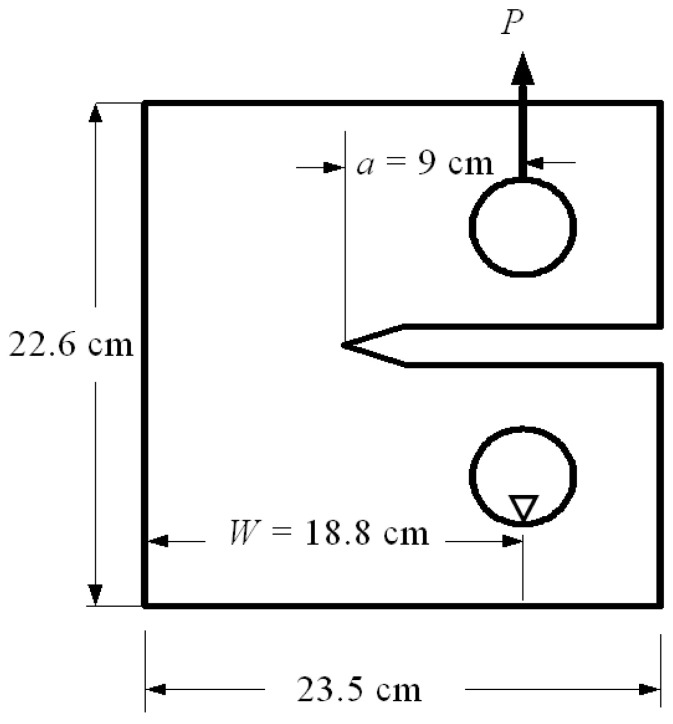
Geometrical dimensions for the compact tension specimen.

**Figure 9:**
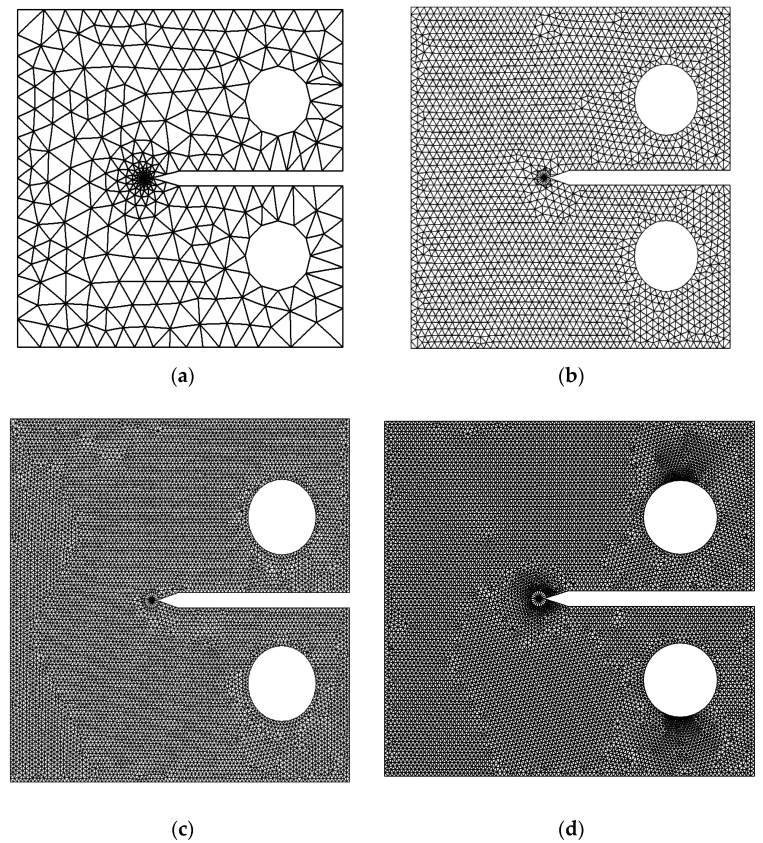
Four different mesh generations with different densities for the compact tension specimen, (**a**) Coarse Mesh, (**b**) medium mesh, (**c**) fine mesh and (**d**) ultra-fine mesh.

The first example was a compact tension specimen, which is a standardized test specimen used in materials testing to measure the fracture toughness of a material. It is primarily used in the field of fracture mechanics to evaluate the resistance of a material to crack propagation. Figure 8 illustrates the dimensions of the compact tension specimen used in the simulation. The problem was investigated using a standard mesh initially, followed by an adaptive mesh that was refined in the vicinity of the crack and the load position. The final mesh employed a significantly higher density compared to the preceding three meshes, as shown in Figure 9. The four different mesh configurations generated element counts of 745, 2754, 4350, and 18,950, individually. The material properties are modulus of elasticity *E* = 70 GPa, Poisson ratio *ν* = 0.33, and fracture toughness *K_IC_* = 26 MPa mm^0.5^.

The analytical first mode of stress intensity factor for this particular geometry can be determined by utilizing the equation provided by Anderson [48], which can be expressed as

(25) $$\begin{array}{l} K_{I}=P\left(2+\frac{a}{W}\right)\left(0.886+4.64\left(\frac{a}{W}\right)-13.32{\left(\frac{a}{W}\right)}^{2}\right.\\ \;\;\;\;\;\;\left.+14.72{\left(\frac{a}{W}\right)}^{3}-5.6{\left(\frac{a}{W}\right)}^{4}\right)/B\sqrt{W}{\left(1-\frac{a}{W}\right)}^{3/2} \end{array}$$

where *P* denotes the applied load, *a* is the crack length, *W* represents the width, and *B* represents the thickness of the geometry.

**Figure 10:**
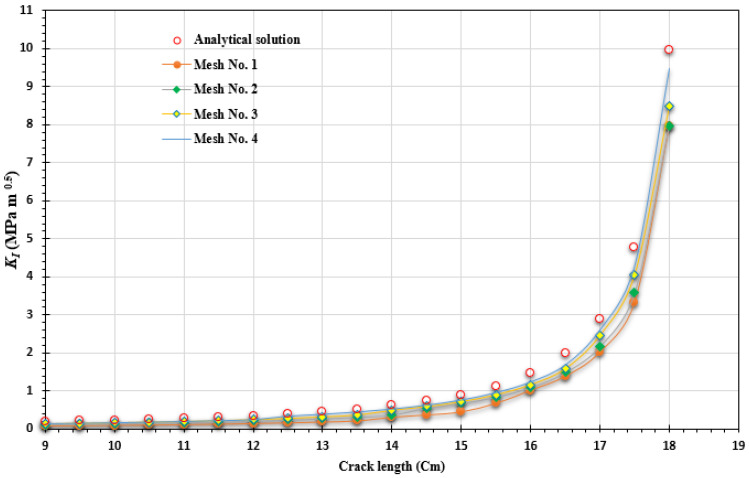
Comparative evaluation of *K_I_* with an analytical solution across different mesh densities for the compact tension specimen.

To assess the accuracy of the predicted results for the first mode of stress intensity factor, obtained from four different types of generated mesh, a comparison was made with the analytical solution presented in Equation (25). This analysis aimed to evaluate how well the predicted values aligned with the analytical solution across varying mesh densities, highlighting the reliability and precision of the results. During the comparisons, a consistent applied load of 8 kN was utilized, along with a geometry thickness of 8.3 mm. These specific values were adopted from the experimental work conducted by Parnas and Bilir [49], who used the same geometry in their experiments. The percentage of error for the four types of mesh configurations, starting from the coarsest to the densest, were as follows: 0.7%, 0.6%, 0.35%, and 0.3%. These comparisons with the analytical solution revealed a decreasing trend in the error percentage as the mesh density increased. The densest mesh configuration exhibited the lowest error percentage, indicating a closer agreement with the analytical solution, as shown in Figure 10. 

**Figure 11:**
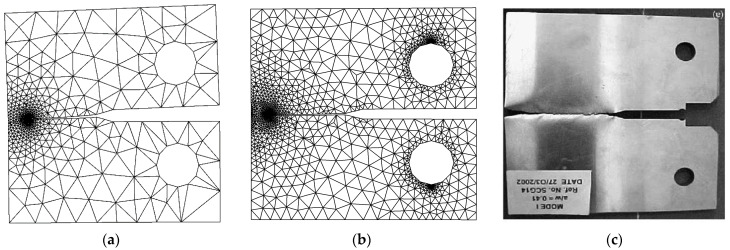
Predicted crack growth path: (**a**) coarse mesh, (**b**) intermediate mesh, (**c**) experimental crack path [50].

To validate the accuracy of the program’s crack growth path prediction, a comparison was made between two predicted paths using coarse and intermediate mesh densities, as well as an experimental crack growth path obtained by Mourad et al. [50]. The comparison demonstrated a distinct agreement between the predicted paths and the experimental results, as depicted in Figure 11. 

It can be observed that the crack growth direction strictly adheres to pure mode I loading. This implies that the crack propagates along a straight path with an angle of zero degrees. Additionally, the values of the second mode of stress intensity factors are nearly negligible. This provides further evidence of the dominance of mode I stress and the straight-line path of crack progression.

The following Table 2 summarizes the computational costs associated with mesh refinement for this example:

### 3.2. Three-Point Bend Specimen

**Figure 12:**
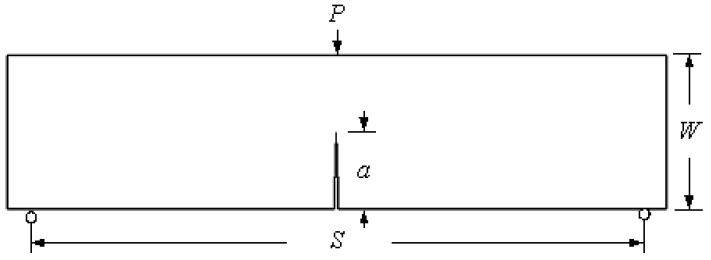
Geometric dimensions of the three-point bend specimen.

The dimensions of the three-point bend geometry are depicted in Figure 12, where the crack length is represented by “*a*”, the span length by “*S*”, and the width by “*W*”. The material properties are modulus of elasticity *E* = 70 GPa, Poisson ratio *ν* = 0.33, and fracture toughness *K_IC_* = 26 MPa mm^0.5^.

Broek [51] provides the analytical formula for calculating the stress intensity factor of the specimen as

(26) $$\begin{array}{l} K_{I}=\frac{PS}{BW^{3/2}}\left[2.9{\left(\frac{a}{W}\right)}^{1/2}-4.6{\left(\frac{a}{W}\right)}^{3/2}+21.8{\left(\frac{a}{W}\right)}^{5/2}\right.\\ \left.\;\;\;\;\;-37.6{\left(\frac{a}{W}\right)}^{7/2}+38.7{\left(\frac{a}{W}\right)}^{9/2}\right] \end{array}$$

where *P* denotes the applied load and *B* represents the specimen thickness.

**Figure 13:**
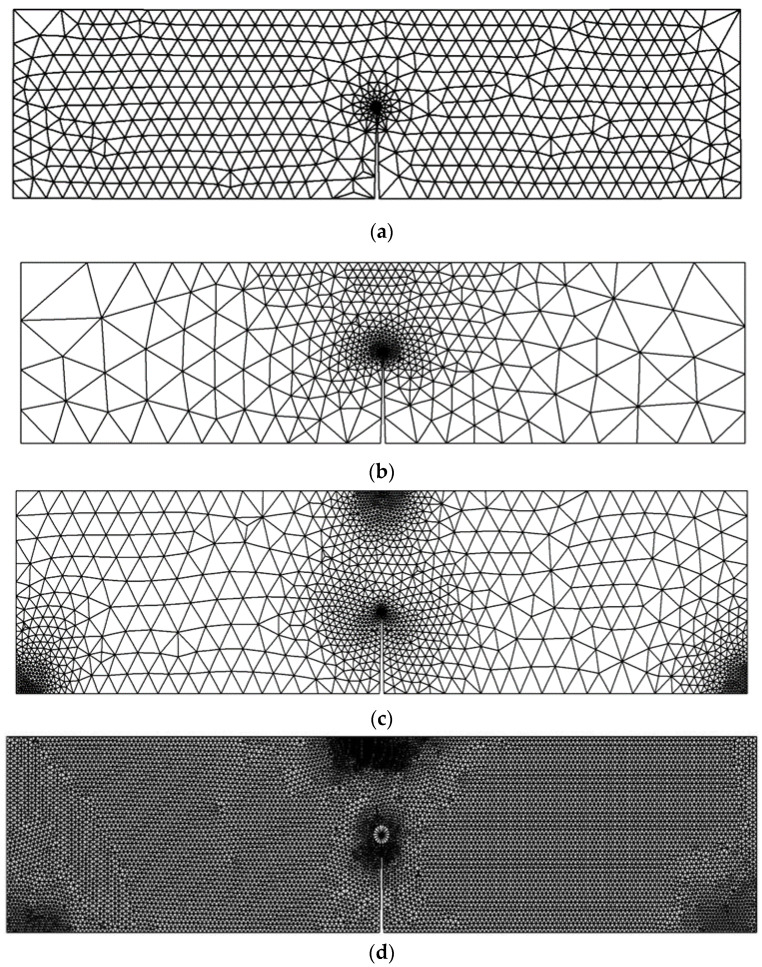
Three different mesh generations with different densities for the three-point bend specimen, (**a**) Coarse Mesh, (**b**) medium mesh, (**c**) fine mesh and (**d**) ultra-fine mesh.

**Figure 14:**
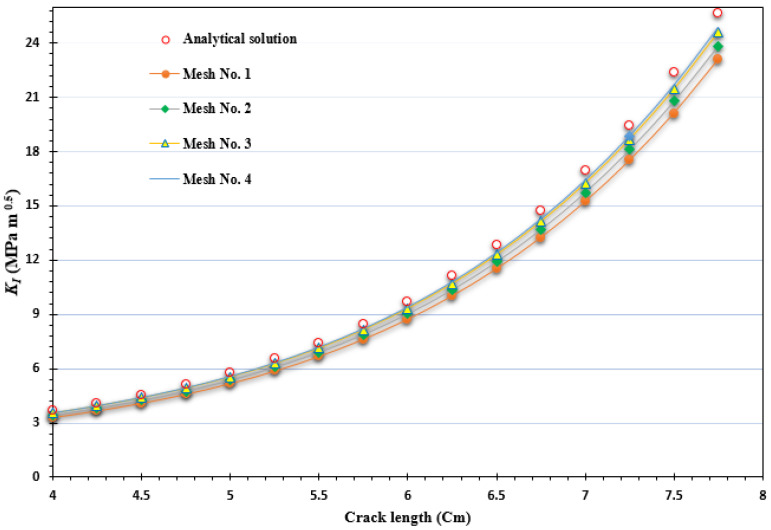
Comparative evaluation of *K_I_* with analytical solution across different mesh densities for the three-point bend specimen.

**Figure 15:**
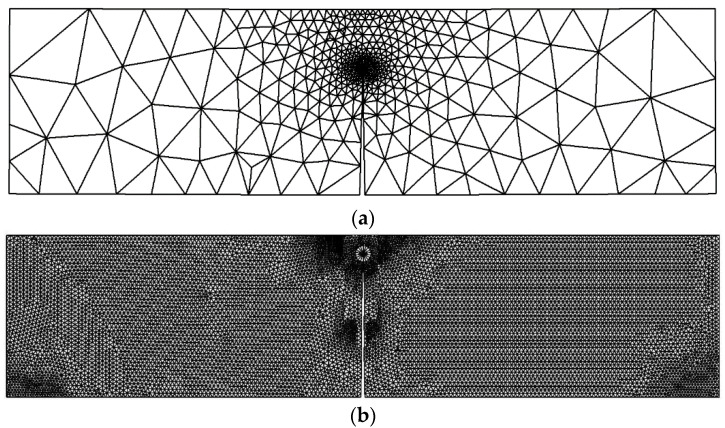
Crack growth path for the (**a**) coarse and (**b**) high-density meshes for the three-point bend specimen.

The problem was explored using three distinct mesh configurations, each characterized by varying densities. In all instances, the mesh was adaptively refined to enhance accuracy near the crack and load position. Notably, the final mesh employed a significantly higher density compared to the preceding two meshes, as clearly illustrated in Figure 13. The specimen had dimensions of *B* = 1 cm, *S* = 32 cm, and *W* = 8 cm, with an initial crack length of *a* = 4 cm, subjected to an applied load of *P* = 9.8 kN. These specific values were adopted from the experimental study reported by Wei and Zhao [52]. The stress intensity factors were computed using three different mesh densities and were compared to the analytical solution given by Equation (26), as shown in Figure 14. This comparison serves to evaluate the accuracy of the calculated SIFs with respect to the established analytical solution. The percentage of error for the three types of mesh configurations, ranging from the coarsest to the densest, were as follows: 0.07%, 0.04%, and 0.03%. These values represent the deviation between the calculated stress intensity factors obtained from the respective mesh configurations and the analytical solution. The decreasing trend in the error percentage indicates an improvement in accuracy as the mesh density increases, with the densest mesh configuration exhibiting the lowest error percentage. Figure 15 illustrates the final crack growth path for both the coarse and high-density meshes.

The crack growth path depicted in the figure demonstrates a pure mode I crack growth, indicating the dominance of mode I fracture behavior.

### 3.3. Three-Point Bending Beam with Three Circular Holes

**Figure 16:**
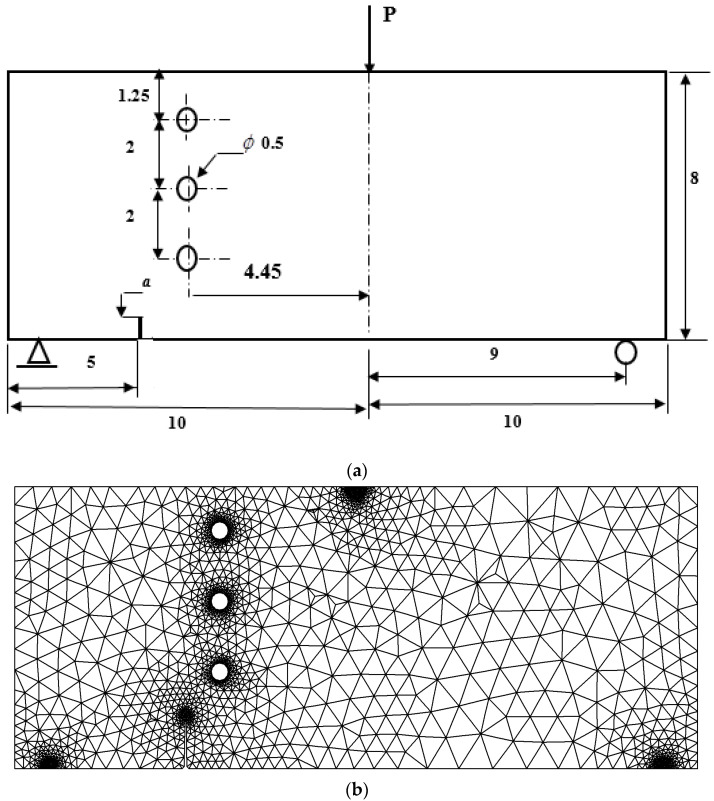

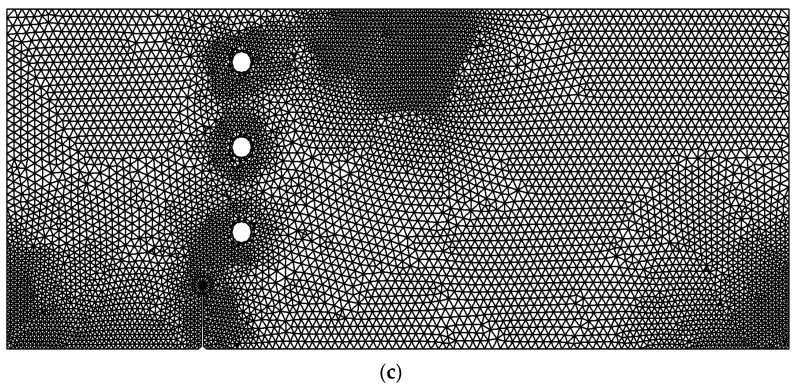
Three-point bending beam with three circular holes (**a**) geometrical configuration and its adaptive mesh (**b**) coarse mesh, and (**c**) ultra-fine mesh.

**Figure 17:**
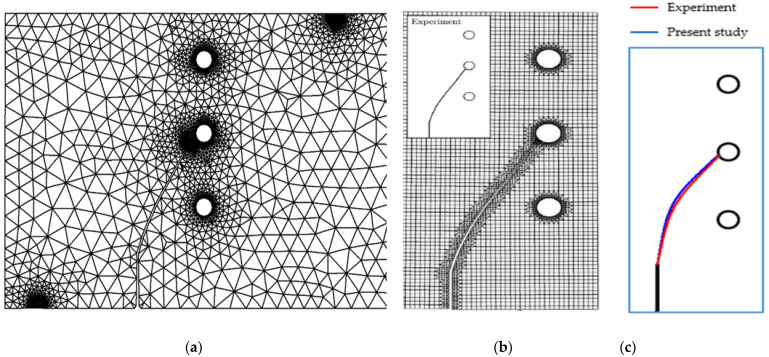
Predicted crack growth trajectory: (**a**) the present study, (**b**) experimental results obtained by [53] and numerical results obtained by [54], and (**c**) present study results compared with the experiment [53].

To assess the precision of the current algorithm in simulating crack growth scenarios, a three-point bending test was modelled, and the resulting path of crack growth was juxtaposed with the experimental outcomes cited in [53]. The geometrical configuration and boundary condition of a three-point bending beam with three circular holes, each having a diameter of 0.5 cm, are visually depicted in Figure 16. Additionally, the figure displays the adaptive mesh used to discretize the beam, demonstrating two different mesh densities. The problem involves a beam made of polymethyl methacrylate (PMMA) with an elastic modulus (*E*) of 2.8 GPa and a Poisson’s ratio (ν) of 0.3. A vertical crack was positioned along the bottom edge of the structure, with its length denoted as “a 1 cm”. Figure 17 depicts the simulated crack growth paths, along with a comparison to the experimental results obtained by Ingraffea [53] and the numerical results obtained by Mohmadsalehi [54], utilizing the conforming to interface structured adaptive mesh refinement (CISAMR) algorithm coupling with ABAQUS software. As can be seen in the figure, the presence of the lower hole had minimal influence on the direction of crack growth. This was because the initial crack was located far away from the lower hole, resulting in negligible interaction. However, the middle hole exerted a stronger influence, causing the crack to be attracted towards it and propagate into the hole. Importantly, the simulation results for these cases demonstrated excellent agreement with the crack shape observed in the three-point bending experiments. This agreement between the simulated and experimental crack patterns reinforces the accuracy and reliability of the simulation methodology. The geometrical dimensions of the crack path in both simulations and experiments are depicted in Figure 17c. The crack paths obtained exhibited a high degree of similarity to those simulated in the current study and experimentally conducted in [53].

### 3.4. Double-Edge Cracked Plate with Two Holes

**Figure 18:**
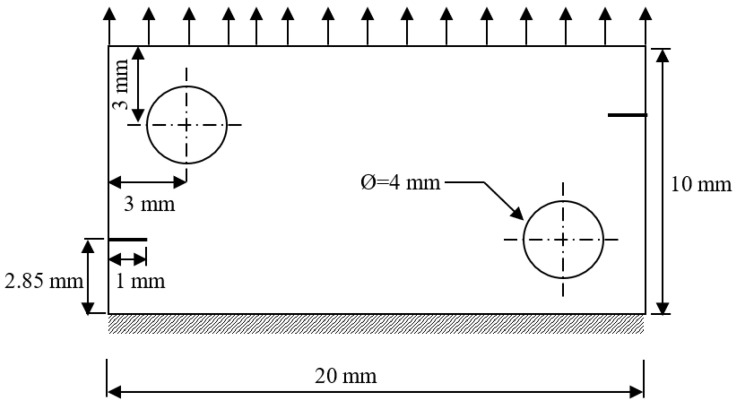
Geometric dimensions of the double edge cracked plate with two holes.

**Figure 19:**
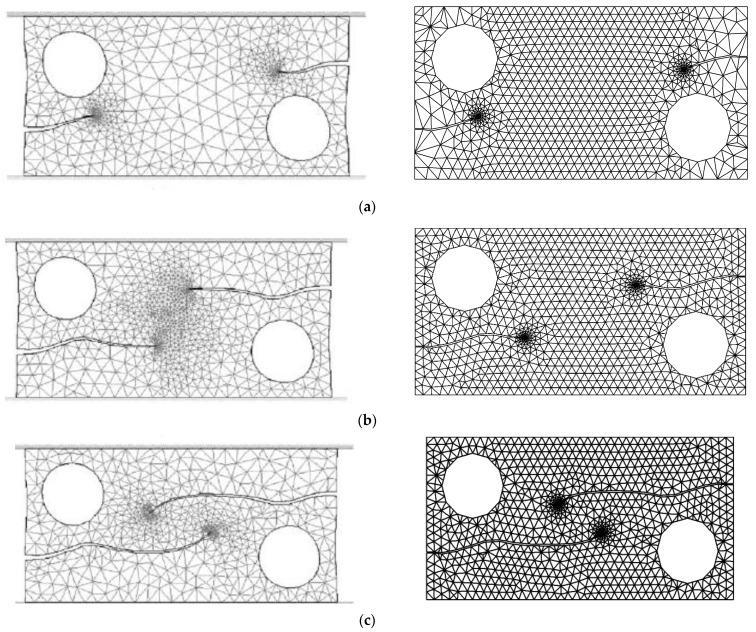

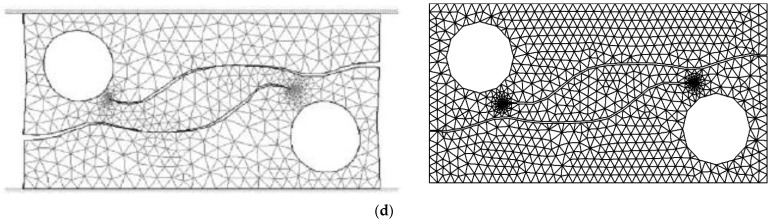
Comparison of crack growth trajectories: simulated results by Bouchard et al. [55] (**Left Side**) and present study results (**Right Side**), (**a**–**d**) represent four different steps of crack growth.

The geometry of the double-edge cracked plate with two holes under the assumption of plane stress is illustrated in Figure 18. The plate is fixed at its lower edge and subjected to a concentrated load at the upper edge. The material properties of the specimen include a Young’s modulus of 98 GPa and a Poisson’s ratio of 0.3. The findings of this research were juxtaposed with the numerical simulation outcomes acquired by Bouchard et al. [55] for a comprehensive comparison. This comparison highlighted four distinct steps of crack progression. Initially, during the first step, each crack was observed to grow towards the closest hole, given that it triggers a decrease in stress, as depicted in Figure 19a. Subsequently, once the crack tip progressed past the hole, its orientation shifted horizontally under mode I loading, as demonstrated in Figure 19b. This alteration is attributable to the change in the stress distribution at the crack tips, as illustrated in Figure 19c. Eventually, in the final phase, the cracks were once again drawn towards the opposite hole, causing them to curve in that direction, as shown in Figure 19d. This entire process highlights the dynamic nature of crack growth and the various factors that influence its direction and propagation. The trajectory of the crack closely resembles the simulation conducted by Bouchard et al. [55], as depicted in this figure.

## 4. Conclusions

This paper introduced a robust algorithm implemented in Visual Fortran that efficiently generates high-quality unstructured triangular meshes for modelling complex two-dimensional crack growth problems. The algorithm successfully addresses the challenges associated with geometric complexity, simulation accuracy requirements, and computational resource constraints. The proposed algorithm successfully incorporates adaptive refinement to predict crack paths with high precision. Moreover, the consistency of the obtained stress intensity factor results with established analytical solutions for standard geometries further validates the algorithm’s accuracy and reliability. The comprehensive details and step-by-step development of the proposed algorithm in the present form provide a valuable tool for researchers to delve deeper into the field of fracture mechanics. By providing a solid foundation, the proposed algorithm stimulates innovation and encourages the advancement of knowledge in this specialized domain.

## Figures and Tables

**Table 1:** Nodal displacement, strain matrices, and element volumes [37].

Application	$d_{i}^{e}$	$B_{i}^{e}$	d*V*
Plane stress	$\left\{\begin{array}{c} u_{i}^{e}\\ v_{i}^{e} \end{array}\right\}$	$\left[\begin{array}{cc} {\left(\frac{\partial N_{i}}{\partial x}\right)}^{e} & 0\\ 0 & {\left(\frac{\partial N_{i}}{\partial y}\right)}^{e}\\ {\left(\frac{\partial N_{i}}{\partial y}\right)}^{e} & {\left(\frac{\partial N_{i}}{\partial x}\right)}^{e} \end{array}\right]$	$t^{e}\;\det\;J^{e}\;d\xi \;d\eta$
Plane strain	$\left\{\begin{array}{c} u_{i}^{e}\\ v_{i}^{e} \end{array}\right\}$	$\left[\begin{array}{cc} {\left(\frac{\partial N_{i}}{\partial x}\right)}^{e} & 0\\ 0 & {\left(\frac{\partial N_{i}}{\partial y}\right)}^{e}\\ {\left(\frac{\partial N_{i}}{\partial y}\right)}^{e} & {\left(\frac{\partial N_{i}}{\partial x}\right)}^{e} \end{array}\right]$	$\det\;J^{e}\;d\xi \;d\eta$

**Table 2:** Computational costs associated with mesh refinement.

Mesh Density	Number of Elements	Number of Nodes	Computational Time
Coarse mesh	1742	3600	12 min
Medium mesh	3641	7423	27 min
Fine mesh	4523	9488	54 min
Ultra-fine mesh	7254	15,362	90 min

## Data Availability

All relevant data are contained in the present manuscript.
